# Raising Awareness for Lung Cancer Prevention and Healthy Lifestyles in Female Scholars from a Low-Income Area in Bogota, Colombia: Evaluation of a National Framework

**DOI:** 10.1007/s13187-017-1246-z

**Published:** 2017-07-06

**Authors:** JF Meneses-Echávez, PA Alba-Ramírez, JE Correa-Bautista

**Affiliations:** 1 Norwegian Institute of Public Health, Oslo, Norway; 2 Centro de Estudios en Medición de la Actividad Física (CEMA), Escuela de Medicina y Ciencias de la Salud, Universidad del Rosario, Bogotá, Colombia

**Keywords:** Skin cancer, Sun protection, Risk factor, Prevention

## Abstract

This study aims to determine the effects of an educational intervention, based on the Colombian guidelines for educational communication in the framework of cancer control, for raising lung cancer prevention-related awareness, and improving healthy lifestyles in female scholars from a low-income area in Bogota, Colombia. Uncontrolled trial conducted in 243 female scholars (mean age 14 years ± 1.5 SD). Two 90 min educational sessions were carried out in March 2015 according to the Colombian guidelines for educational communication in the framework of cancer control. Posters and other educational materials were created by scholars after the intervention. All participants completed a self-reported questionnaire—The Cancer Awareness Measure—at pre and post-intervention, as well as 1, 3, and 6 months after the intervention. Smoking prevalence (8.2% at baseline) was reduced by 3.7% at 6 months follow-up (*p* < 0.005). The scholars exhibited low to moderate awareness of both warning signs and risk factors for lung cancer at baseline. These variables showed statistically significant improvements at 6 months follow-up (*p* < 0.005). Similar improvements were also found for physical activity, high-fat diet, and fruits and vegetable intake. This evaluation of the Colombian guidelines for educational communication in the framework of cancer control raised awareness towards lung cancer prevention, reduced smoking, and improved other healthy-lifestyle-related factors in a group of female scholars from a low-income area in Bogota, Colombia. Further randomized controlled studies are needed.

## Introduction

Lung cancer is a leading cause of cancer death and the second most common cancer in both men and women (not counting skin cancer) [1]. The incidence of lung cancer and smoking is higher in developed countries, where western lifestyles are more prevalent [1, 2]. Smoking is associated with more than 30% of all cancer deaths, including 80% of all deaths attributed to lung cancer [3, 4]. Some biological mechanisms such as DNA demethylation [5] and the production of tumor antigen-specific antibodies [6] can support the link between smoking and lung cancer.

Most of cancer types are preventable by adopting healthy lifestyles [7]. In a recent meta-analysis of 28 observational studies, Brenner and colleagues [8] found an inverse association between recreational physical activity and lung cancer risk (relative risk (RR), 0.76; 95% confidence interval (CI), 0.69–0.85). Furthermore, physical activity levels are also associated with lower lung cancer mortality (low physical activity, hazard ratio (HR) 0.80 (0.69–0.92); medium physical activity, HR 0.68 (0.59–0.80); and high physical activity, HR 0.78 (0.66–0.93)) [9]. Similar evidences have been published regarding fruits and vegetables intake (RR 0.86; 95% CI 0.78–0.94) [10].

Adolescence is a critical period when lifelong habits are established, and is also when the onset of smoking takes place [11, 12]. These factors are clearly integrated in school-based programs for smoking prevention and healthy lifestyles promotion [13]. School-based programs for smoking prevention have proven favorable effects in different groups [14, 15]. Most of these effects have been attributed to different modifies such as behavioral intentions, knowledge, social influences, and beliefs [16, 17].

In the Colombian context, there are scarce evidences about educational interventions for both smoking and lung cancer prevention, whereas the benefits of the national guidelines for educational communication in the framework of cancer control remain unknown [18]. The current agenda for cancer control in Colombia emphasizes on the need of community-based research for both detection and prevention [19]. This study aims to evaluate the effects of an educational intervention for raising awareness for lung cancer prevention and healthy lifestyles in a group of female scholars from a low-income area in Bogota, Colombia.

## Methods

### Design

Uncontrolled trial had repeated measurements at 1, 3, and 6 months post-intervention.

### Participants

We selected a convenience sample of 243 female adolescents (10–17 years old), students from a public school in a low-income area in Bogota, Colombia. The purpose of study, interventions, and dates were explained in the classroom to the eligible students. Those who referred pregnancy, breastfeeding, or previous participation in a similar educational intervention were excluded.

### Ethics

Ethical approval to conduct this study was obtained from the ethics committee of the Our Lady of the Rosary University (Reference No. 306), after approval of the research protocol. Written informed consent was taken for each participant.

### Data Collection

#### Lung Cancer Awareness: Warning Signs and Risk Factors

We used the Cancer Awareness Measure (CAM) [20–22], which was developed and validated in 2007–2008 by the Cancer Research UK, the University College London, the King’s College, and Oxford University. The CAM is a validated questionnaire planned to assess awareness of cancer among the general population. The CAM collects information about warning signs, help-seeking, and risk factors of the most common cancers. CAM developers suggested the questionnaire can be used at national, regional, and local levels to monitor/track awareness over time, compare between groups, identify information needs, and monitor the impact of awareness-raising interventions. Responses were measured using the nominal scale of “Yes” and “No.”

#### Healthy Lifestyle

Scholars’ lifestyles were evaluated by using the Spanish version of the Behavioral Risk Factor Surveillance System published by the Center for Disease Control and Prevention (CDC) [23]. The following domains were evaluated: smoking, high fat diet, physical activity (>150 min/week), and fruits and vegetables intake. Weekly use/consumption was measured using the nominal scale of “Yes” and “No.”

#### Questionnaire Validity

All modules were translated into Spanish by one researcher and back translation was conducted and checked by one independent bilingual translator to ensure equivalence. The final version of the questionnaire was then piloted in a subgroup of 50 students who provided feedback regarding feasibility, clarity, and understanding. No lingual difficulties were identified.

The questionnaire was completed by all participants under supervision of one researcher (PAAR), as suggested by the Cancer Research UK [20]. Cronbach’s alpha was used to assess the internal consistency, and Pearson’s correlation test was used to evaluate the test- retest reliability over a 7-day interval. Sensitivity to change is interpreted with the study results (i.e., changes scores).

### Educational Intervention

The educational intervention was developed in accordance with the national guidelines for educational communication in the framework of cancer in Colombia [18], by incorporating the use of clear language, flexible and understandable vocabulary. In order to facilitate the adherence and completion, the intervention contents were articulated into the school curriculum. The two 90-min educational sessions were carried out in March 2015 emphasizing on the normal lung, lung cancer warning signs and risk factors, smoking, and the role of healthy lifestyles on lung cancer prevention (Table 1). One researcher and teacher (PAAR) supervised both sessions and procedures. The intervention content was discussed and approved by all authors. The pedagogical resources were videos, presentations, and open discussions. The students prepared various posters and placed them on the most crowded areas of the school. At the end of the sessions, each participant received a copy of the educational content provided. The scholars did not receive any other educational information in their school curriculum, apart from the two 90-min sessions of the intervention, related to the aim of this study. Follow-up assessments were undertaken at 1, 3, and 6 months after the intervention.

**Table 1:** Educational intervention for lung cancer prevention

Educational intervention for lung cancer prevention through healthy lifestyles, key components
• Introduction to healthy lifestyles and cancer prevention
• Review of the learning objectives
• Normal lung (basic anatomy and physiology)
• Warning signs for lung cancer
• Lung cancer risk factors (i.e., modifiable and non-modifiable)
• Symptoms of lung cancer
• Early detection of lung cancer
• Treatment options of lung cancer

### Statistical Analysis

Descriptive statistics were used to present the sociodemographic characteristics of students (i.e., frequency, percentage, mean, and standard deviation). A multilevel regression model was used to estimate the change coefficients in the different scores with respect to pre-intervention measurements; each measurement was then assumed as a hierarchical source of variability [24]. Alpha levels of *p <* 0.05 were considered as significant. The data were analyzed using the Stata version 22.0.

## Results

1. Questionnaire performance and reliability

Cronbach’s alpha provided scores over 0.7 for all items in the questionnaire, which suggested a strong level of internal consistency. Similarly, Pearson’s correlation test showed strong the test-retest reliability (over 0.72) for all items (*p* < 0.05) over a 7-day interval.

2. Sociodemographic characteristics of study participants

A total of 243 female students participated in the educational intervention and received the whole content. Their mean age was 14 years ± 1.5 standard deviations (SD) (range 12–17). All students are residents in a low-income area in south Bogota, Colombia. Around half of the participants live with both parents and less than half of the parents have completed academic studies (Table 2).

**Table 2:** Sociodemographic characteristics of study participants (*n* = 243)

Characteristics	*n*	Percentage
Age (years)		
<15	181	74.5
>15	62	25.5
Home composition		
Both parents	138	56.8
Only father	20	8.2
Only mother	85	35
Father’s education		
Primary school	36	14.8
High school	100	41.2
University	107	44
Mother’s education		
Primary school	27	11.1
High school	97	39.9
University	119	(49)

3. Effects of the educational intervention on awareness of warning signs for lung cancer

Baseline measurements revealed low awareness of the warning signs for lung cancer. Awareness of all warning signs for lung cancer showed exponential increases across all measurements (*p* < 0.0001). See Table 3.

4. Effects of the educational intervention on student’s awareness of lung cancer risk factors

**Table 3:** Effects of the educational intervention on awareness of warning signs for lung cancer

Warning sign for lung cancer	Pre^a^	Post	1 month	3 months	6 months
	*n* (%)	*n* (%)	*n* (%)	*n* (%)	*n* (%)
		*p* value	*p* value	*p* value	*p* value
Unexplained lump or swelling	97	170	160	136	188
	39.9	30.0	25.9	16.0	37.4
		(0.000)^b^	(0.000)^b^	(0.000)^b^	(0.000)^b^
Persistent unexplained pain	138	195	143	159	179
	56.8	23.4	2.0	8.6	16.8
		(0.000)^b^	(0.624)	(0.040)^b^	(0.000)^b^
Unexplained bleeding	134	223	180	188	203
	55.1	36.6	18.9	22.2	28.3
		(0.000)^b^	(0.000)^b^	(0.000)^b^	(0.000)^b^
Persistent cough or hoarseness	83	100	114	122	152
	34.2	06.9	12.7	16.0	28.3
		(0.109)	(0.003)^b^	(0.000)^b^	(0.000)^b^
Persistent change in bowel or bladder habits	96	161	155	138	177
	39.5	26.7	24.2	17.2	33.3
		(0.000)^b^	(0.000)^b^	(0.000)^b^	(0.000)^b^
Persistent difficulty swallowing	53	80	92	102	138
	21.8	11.1	16.0	20.1	34.9
		(0.009)^b^	(0.000)^b^	(0.000)^b^	(0.000)^b^
A sore that does not heal	66	208	131	129	141
	27.2	58.4	26.7	25.9	30.8
		(0.000)^b^	(0.000)^b^	(0.000)^b^	(0.000)^b^
Unexplained weight loss	78	202	132	123	160
	32.1	51.0	22.2	18.5	33.7
		(0.000)^b^	(0.000)^b^	(0.000)^b^	(0.000)^b^

The percentages reflect the score change with respect to pre-intervention values

^a^Affirmative responses to each variable

^b^Statistically significant differences

More than half of the students identified smoking as the main risk factor for lung cancer, and this outcome increased by 12.3% at 6 months post-intervention (*p* < 0.0001). Similar patterns were observed on the awareness of secondhand smoke. Students’ awareness for other behavioral risk factors such as sedentarism and fruits and vegetables intake showed similar exponential increases. The educational intervention increased the recommendation by students of physical exercise for both prevention and treatment of lung cancers up to 6 months follow-up (Table 4).

**Table 4:** Effects of the educational intervention on student’s awareness of lung cancer risk factors

Lung cancer risk factors	Pre^a^	Post	1 month	3 months	6 months
	*n* (%)	*n* (%)	*n* (%)	*n* (%)	*n* (%)
		*p* value	*p* value	*p* value	*p* value
What is lung cancer?	124	225	214	226	230
	51	41.5	37.0	41.9	43.6
		(0.000)^b^	(0.000)^b^	(0.000)^b^	(0.000)^b^
Smoking	183	213	201	210	213
	75.3	12.3	7.4	11.1	12.3
		(0.000)^b^	(0.025)^b^	(0.001)^b^	(0.000)^b^
Secondhand smoke	165	209	176	197	208
	67.9	18.1	4.5	13.1	17.6
		(0.000)^b^	(0.217)	(0.000)^b^	(0.000)^b^
Low intake of fruits and vegetables	40	96	75	60	97
	16.5	23.0	14.4	8.2	23.4
		(0.000)^b^	(0.000)^b^	(0.045)^b^	(0.000)^b^
Sedentarism	59	120	85	75	132
	24.3	25.1	10.6	6.5	30
		(0.000)^b^	(0.013)^b^	(0.126)	(0.000)^b^
Would you recommend exercise for lung cancer prevention?	209	238	237	229	222
	86	11.9	11.5	8.2	5.3
		(0.000)^b^	(0.000)^b^	(0.000)^b^	(0.016)^b^
Would you recommend exercise for patients with lung cancer?	200	218	223	215	220
	82.3	7.4	9.4	6.1	8.2
		(0.008)^a^	(0.001)^a^	(0.028)^a^	(0.003)^a^

The percentages reflect the score change with respect to pre-intervention values

^a^Affirmative responses to each variable

^b^Statistically significant differences

5. Effects of the educational intervention on healthy lifestyles for lung cancer prevention

The educational intervention decreased weekly self-report of smoking at 6 months after the intervention (3.7% decrease; *p* < 0.05). Improvements were also observed on the practice of physical activity and the intake of high-fat food, vegetables, and fruits. See Table 5.

**Table 5:** Effects of the educational intervention on healthy lifestyles for lung cancer prevention

Domains of lifestyle (weekly use)	Pre^a^	Post	1 month	3 months	6 months
	*n* (%)	*n* (%)	*n* (%)	*n* (%)	*n* (%)
		*p* value	*p* value	*p* value	*p* value
Smoking	20	8	9	4	11
	8.2	−4.9	−4.5	−6.5	−3.7
		(0.007)^b^	(0.013)^b^	(0.000)^b^	(0.043)^b^
High fat diet	162	148	150	145	158
	66.7	−5	−4	−6	−1.6
		(0.164)	(0.232)	(0.091)	(0.691)
Physical activity (>150 min/week)	159	174	158	181	166
	65.4	6.1	−0.4	9.0	2.8
		(0.141)	(0.922)	(0.031)^b^	(0.492)
Fruits intake	170	195	186	201	187
	70	10	6.5	1.2	6.9
		(0.007)^b^	(0.082)	(0.001)^b^	(0.065)
Vegetables intake	195	200	202	208	209
	80.2	2.0	2.8	5.3	5.7
		(0.538)	(0.388)	(0.109)	(0.084)

The percentages reflect the score change with respect to pre-intervention values

^a^Affirmative responses to each variable

^b^Statistically significant differences

## Discussion

### Main Findings, Agreements, and Disagreements with Other Studies

Our findings demonstrated an educational intervention, based on the national guidelines for educational communication in the framework of cancer control, raised students’ awareness of both warning signs and risk factors for lung cancer, as well as healthy lifestyles up to 6 months follow-up.

#### Awareness of Warning Signs and Risk Factors for Lung Cancer

Similar findings have been reported in a controlled study in four UK schools, where the authors assessed the effectiveness of an educational intervention delivered by Teenage Cancer Trust [25]. Besides, fairly similar awareness of lung cancer risk factors was observed among both British and Colombian adolescents [25]; while higher levels of awareness have been found in German high school students, 92% of the German scholars identified smoking as a risk factor for lung cancer [26].

The present educational intervention raised female scholars’ awareness on lung cancer prevention through healthy lifestyles. Our findings align with those found in a similar single-group, pretest/posttest program evaluation of a teacher-led version of the St. Jude Cancer Education for Children Program (SJCECP) in fourth-grade students from 10 local schools in Memphis area [27]. Moreover, other similar educational approach, the program “I do not smoke, I exercise”—a theory-based smoking prevention program that promotes exercise as an alternative of smoking—yielded positive effects on adolescents’ awareness for lung cancer prevention in Greek secondary schools [28]. The authors highlighted the importance of integrating physical activity into smoking prevention strategies, especially to improve students’ attitudes towards smoking and awareness about the health consequences of smoking [28].

### Healthy Lifestyles for Lung Cancer Prevention

In spite of some lack of statistical significance, our educational intervention improved healthy lifestyles in the students. The students in this study exhibited significant reductions in smoking throughout the follow-up periods with a 3.7% reduction at 6 months. These effects align with those reported in a recent Cochrane review [29], which also found significant reductions in smoking prevalence for school-based programs that included social competences and social influences curricula (odds ratio 0.88; 95% CI 0.82 to 0.95). Similar effects on smoking have also been reported from Latino-American countries [30]. It has been recently suggested that school-based smoking prevention curricula programs can provide larger results (12% reduction) in never-smokers age 5–18 [29]. Both social competences and influences must be incorporated into such interventions, even if there is no evidence on these factors in Latino-American countries [29, 31].

The current educational intervention improved the practice of physical activity (>150 min/week) in the students evaluated. Comparable findings have been communicated in a Cochrane review (44 studies involving 36.593 children and adolescents), which concluded that educational interventions combining printed educational materials and changes to the school curriculum that promote physical activity during school hours can lead to positive effects in increasing duration of physical activity from 5 to 45 min more per day and other outcomes such as the time spent watching television from 5 to 60 min less per day, and physical fitness [32]. Considering some context-related differences, we are able to recommend school-based interventions for the promotion of physically active behaviors.

Our improvements in fruit and vegetables intake are similar to those published by Evans and collaborators [33] in 2012 summarizing evidences from 27 school-based programs involving 26.361 children, who reported increases of nearly 0.25 portions of fruit and vegetable daily intake. We did not evaluate the number of portions.

### Strengths and Weaknesses

This study is the first evidence in evaluating the Colombian guidelines for educational communication in the framework of cancer control. We followed international recommendations when constructing and evaluating this education intervention, which has been reflected in the use of the CAM questionnaire for collecting the data; similar British studies do so [27]. It is also important to highlight the relevance of involving low-income groups in educational interventions against cancer, since most of the massive campaigns in the country do not reach these individuals [18, 19]. However, the lack of a random assignment, probabilistic sampling methods, and a control group preclude our potential to draw stronger conclusions about the effects of our educational intervention.

### Implications for Research

The study of adolescents’ awareness of lung cancer risk factors, as well as other cancers, will benefit from further experimental research. Well-conducted randomized controlled trials (RTCs) can provide more rigorous evidence about the effects of educational interventions in improving females’ potential for lung cancer prevention through healthy lifestyles. Such RCTs must incorporate large sample sizes and adequate methods for allocation concealment, sequences generation, and outcome measurement. The use of objective measurements for lifestyle-related outcomes is also warranted.

Community-level influences in scholars’ lifestyles were not measured in this study. The Travis Country CATCH trial concluded that community involvement in school-based obesity prevention programs in undeserved population could enhance program outcomes, such as scholars’ behaviors, knowledge, and perceptions towards healthy living [34]. In line with this evidence, we recommend that further research should facilitate and sustain a dynamic community involvement in school-based programs for cancer and other non-communicable conditions.

### Implications for Practice

The results from this study are articulated with the priorities of the cancer risk control and prevention strategies of the Colombian ministry of health [18, 19] and those from other international authorities. We recommend a detailed analysis of the applicability of our study findings since most of the current evidence comes from high-income countries, which precludes its potential for transferability to the Colombian context.

## Conclusion

An educational intervention based on the Colombian guidelines for educational communication in the framework of cancer control raised awareness for lung cancer prevention and improved healthy lifestyles in female scholars from a low-income area in Bogota, Colombia. Further randomized-controlled studies are warranted.
